# Behavior Change Around an Online Health Awareness Campaign: A Causal Impact Study

**DOI:** 10.3389/fpubh.2022.857531

**Published:** 2022-06-23

**Authors:** Victor Suarez-Lledo, Yelena Mejova

**Affiliations:** ^1^Department of Biomedicine, Biotechnology and Public Health, University of Cadiz, Cadiz, Spain; ^2^Computational Social Science DataLab, University Research Institute on Social Sciences, University of Cadiz, Cadiz, Spain; ^3^ISI Foundation, Turin, Italy

**Keywords:** health informatics, health interventions, Twitter, social media, mental health, eating disorders

## Abstract

National Eating Disorders Association conducts a NEDAwareness week every year, during which it publishes content on social media and news aimed to raise awareness of eating disorders. Measuring the impact of these actions is vital for maximizing the effectiveness of such interventions. This study is an effort to empirically measure the change in behavior of users who engage with NEDAwareness content, and compare the detected changes between campaigns in two different years. We analyze a total of 35,895 tweets generated during two campaigns of NEDAwareness campaigns in 2019 and 2020. In order to assess the reach of each campaign, we consider the users participating in the campaigns and their number of followers, as well as retweeting engagement. We use the Linguistic Inquiry and Word Count (LIWC) text modeling and causal impact analysis in order to gauge the change in self-expression of users who have interacted with the NEDAwareness content, compared to a baseline group of users. We further enrich our understanding of the users by extracting gender information from their display names. We find that, despite large media corporations (such as MTV and Teen Vogue) participating in the campaign, it is governmental and nonprofit accounts who are among the accounts that attract the most retweets. Whereas the most influential accounts were well-connected in 2019, the 2020 campaign saw little retweeting between such accounts, negatively impacting the reach of the material. Both campaigns engaged women at around 40% and men 17%, supporting previous research showing women to be more likely to share their experiences with eating disorders. Further, women were more likely to mention other health topics within the 15 days of the intervention, including pregnancy and abortion, as well as depression and anxiety, and to discuss the developing COVID pandemic in 2020. Despite the positive message of the campaign, we find that the users who have engaged with this content were more likely to mention the linguistic categories concerning anxiety and risk. Thus, we illustrate the complex, gender-specific effects of NEDAwareness online health intervention campaign on the continued self-expression of its audience and provide actionable insights for potential improvement of such public health efforts.

## 1. Introduction

Worldwide, eating disorders have been steadily increasing, from 3.5% in 2000–2006 to 7.8% in 2013–2018 (1). Depending on which groups, such as young adults, the incidence of eating disorders is higher (2). Characterized by a severe disturbance in eating behavior and body weight (3), the three most common disorders are anorexia nervosa, bulimia nervosa, and binge eating disorder. There have also been recognized other “disordered eating” behaviors and ideation which do not meet the full criteria for a disorder, but which impact millions of people's lives (4–6). In addition, low self-esteem and other psychological factors are related to eating disorders and media consumption. For example, the literature demonstrates the existence of a relationship between exposure to media cultivating ideals of beauty impacts on body image, eating disorders and low self-esteem (2).

In line with this, social media is a specific type of media where interconnectivity predominates. A problematic use of these platforms is related to decreased self-esteem and mental well-being (7). Social media has been shown to exacerbate these conditions by emphasizing Western culture's obsession with idealized body shape and diet, and by promoting weight-management advise that reinforces cycles of weight loss and regain, exercise avoidance, and anxiety (8). Responding to these trends, the Health at Every Size (HAES) framework that promotes self-acceptance has been used to promote healthier discourse around body image, including on social media and beyond (9).

Social media, and in particular Twitter, have become a source for monitoring health-related perceptions (10). These social platforms have gained wider participation among health information consumers from all social groups regardless of gender or age (11). Much of the current literature is focused on the study of this phenomenon. A great amount of these studies focus on the effects of Twitter as a source of health information or misinformation. With regard to the latter, these studies can be grouped into six main themes: vaccines, drugs and smoking, non-communicable diseases, pandemics and communicable diseases, eating disorders, and medical treatments and interventions (12). With regard to eating disorders, the social media platforms are a meeting point for the dissemination of pro-eating disorder tips (2, 13). These online communities resemble echo chambers where members are selectively exposed to the content they want to see. This echo chamber effect could explain why online campaigns have such a limited effect and often increase polarization, reinforcing proximate views in favor of eating disorders (14).

Health professionals and organizations are also using this medium to disseminate health-related knowledge on healthy habits and medical information for disease prevention, as it represents an unprecedented opportunity to increase health literacy, self-efficacy, and treatment adherence among populations (15, 16). The National Eating Disorders Association (NEDA) is a nonprofit organization dedicated to supporting individuals and families affected by eating disorders that focuses on prevention and access to quality care. Yearly, at the end of February, it conducts the “NEDAwareness” campaign in order to raise awareness, help people to connect, and promote resources for those affected by eating disorders (17). Other similar health awareness campaigns have been conducted and studied on Twitter, including #WorldCancerDay (18), #WakeUpWeightWatchers (19), and #MyTipsForMentalHealth (20). Although such online health interventions are becoming prevalent (21), skepticism persists on the efficacy of health awareness campaigns to change the behavior of the target populations (22), necessitating quantitative case studies of this popular form of intervention.

In previous literature, communities around pro- and anti- eating disorders have been examined on Flickr (23), Tumblr (24) and Instagram (25). Most of these works, however, measure the interaction of the audience with each other or the campaign material, failing to follow up on the potential changes in behavior after the intervention. A notable exception is the measurement of whether those posting to pro-anorexia Flickr communities continue to do so after being exposed to anti-anorexia content (23). The study finds that, unlike the intended effect, these users would post to pro-anorexia communities for a *longer* period of time, adding to the concerns over the efficacy of online interventions. Recently, the tweets around the NEDAwareness campaign have been compared to those around the Wake Up Weight Watchers campaign, finding that the “awareness initiatives generated a greater number of eating disorder-related tweets than those spontaneously posted,” but that they “failed to increase the frequency of tweets containing medical content, personal testimonies of recovery, or offers for treatment” (19). This study also finds that a third of the studied tweets were labeled as personal testimonies, illustrating social media's role as an outlet for self-expression. Unlike previous works, it is the aim of this case study to *measure the change in self-expression of social media users who have interacted with the eating disorders campaign material*.

This study's methodology concerns several levels of comparison. First, we study the self-expression behavior of Twitter users who have interacted with NEDAwareness content from a period before to a period after the interaction in order to capture the changes over time. Second, we compare this change to a baseline set of users who have posted on health-related topics in the past, but who have not interacted with NEDAwareness content. Third, we compare these changes in behavior across 2 years—2019 and 2020—in order to ascertain the stability of the results. To measure the behavior change, we utilize the framework for the event outcome analytics proposed by Olteanu et al. (26) and Kıcıman and Thelin(27), which has been used to gauge the impact of alcohol use in students (28) and psychopathological effects of psychiatric medication (29). Fourth, we compared the differences by gender to see if the differences found in other studies on online campaigns are present (30). In particular, we examine the variability of the campaign's impact in terms of the volume of associated posts, their potential reach, and the effect on user self-expression after campaign engagement. We conclude with a discussion of actionable insights for the design of public health interventions for eating disorders in particular, and on social media in general.

## 2. Methods

### 2.1. NEDAwareness Week Data Collection

The data used in this study spans 2 years of NEDAwareness campaigns, each 1 week long: February 25–March 3, 2019 [collected previously in Mejova and Suarez-Lledó (31)] and February 24–March 1, 2020. To collect the tweets associated with the campaigns, we used the Twitter Streaming API with the following keywords: NEDAstaff, NEDAwareness, NEDA, ComeAsYouAre, SOSChat (compiled with the assistance of NEDA staff). The first campaign in 2019 resulted in 19,432 tweets captured from 10,773 unique users, and the 2020 campaign had 16,463 tweets from 10,402 users (see Table 1 for summary statistics). In order to profile the user behavior, we then attempted to collect the “historical” tweets of users captured in both datasets using the Twitter API call that allows the collection of the most recent 3,200 tweets for each user. We collected these on 21/04/2019 and 16/03/2020 and got 17,336,389 and 4,538,394 tweets, respectively.

**Table 1:** Summary statistics of the 2019 and 2020 datasets.

**Metric**	**2019**	**2020**
Time span	February 25–March 3, 2019	February 24–March 1, 2020
NEDA tweets and retweets	19,432	16,463
NEDA users	10,773	10,402
Histories of NEDA users	17,336,389	4,538,394
NEDA selected users	1,746	431
Baseline selected users	2,991	6,743

### 2.2. Campaign Reach

To assess the reach of each campaign, we considered the users captured in the data. Note that it is impossible for us to distinguish whether each of the tweets were coordinated with the campaign, so we treat all retweets as potential interactions with the campaign material. To estimate the potential pool of viewers of the published content, we examined the number of the followers of each user, as provided by the Twitter API. Though note that, when we add all follower numbers, we cannot exclude overlap of users who may follow several accounts in our data, making the sum as a hypothetical maximum. We also examined the retweet networks of the two campaigns, where a node is a user and an edge signifies at least one retweet of one user's tweet by another. We ran a community detection algorithm to find sets of users most important in collaborating and promoting the content.

### 2.3. Behavioral Impact

In order to measure the impact of the NEDAwareness campaigns, we used the text modeling and statistical analysis of the tweet content before and after engaging with the campaign, for the whole dataset and for each of the genders separately, as described below.

#### 2.3.1. Timeline Partitioning

We began by partitioning the posting timelines of each user into 15 days before and 15 days after an interaction with the NEDAwareness content. Following previous work on health behavior change (32), we define “day 0” as the first time a user engages with NEDA content by retweeting it or posting a related tweet. We consider only the users who have sufficient posting information in both before and after periods, putting a threshold of at least 3 tweets in each period, and call these users the “target users.” This filtering resulted in 1,746 users in 2019 and 431 users in 2020. Note here that the smaller number of selected users in 2020 indicates a lesser amount of posting activity in that time period.

Further, we considered a “baseline” set of users, such that they can be compared to those engaging with NEDA content. To do this, we sampled users who have tweeted in the same spans of time on any diet or health related words^1^, and collected their historical tweets. Lacking a theoretical constraint on the properties of users in NEDAwareness dataset, we do not use matching for this process. The baseline datasets contain 539,844 tweets of 2,991 different users in 2019 and 1,053,803 tweets of 6,743 users in 2020. For these users, we selected the first day of each NEDAwareness campaign as the day 0, such that the time span of the data is roughly similar to that of the target users.

#### 2.3.2. Gender

We enriched our understanding of the users captured in this data by extracting gender information from their display names. To do this, we used a combination of baby name dictionaries published by the National Records of Scotland (33) and United States National Security (34) and a large collection of Google+ accounts from previous literature (35). All together, these resources list 106,683 names with corresponding gender information. We also crafted rules for the detection of honorifics such as “Mrs.” or “Mr.” which correspond to a gender. Secondly we removed special characters from the user names and looked for each of the words in the extensive lists of first names with associated genders. Note that if the name appeared both in male and female lists, it was not labeled with gender due to ambiguity. Unfortunately we did not find many occurrences of self-identified gender information (for instance in specifying the preferred pronouns), thus we acknowledge that this approach may mislabel atypical gender identities.

To gauge its accuracy, we manually selected 100 users from each year and labeled their genders, resulting in an estimated accuracy of 78% (for 2019) and 80% (2020). The manual check for user genders involved reading the user screen name and their self-description. The tweets were not considered. Both authors of the paper participated in the annotation. The procedure consisted of verifying that the first name was properly located in the screen name and that it could plausibly refer to a person of the given gender. For instance, the female label produced for “Alien T alien,” matching Alien to a female name, was labeled as incorrect, whereas the female label for "Brigitte Lanteri" was judged as correct. The correctness of the labels was determined using domain knowledge of internet slang and linguistics.

#### 2.3.3. Text Modeling

We model the self-expression of the users captured in this study using Linguistic Inquiry and Word Count (LIWC) dictionary (36), a dictionary of grammatical, psychological, and content word categories widely used for modeling demographic and psychological characteristics, including mental health (37). It contains 72 lexical categories grouped into (1) standard linguistic process, (2) psychological process, (3) relativity, and (4) personal concerns. We do not consider categories dealing with basic grammar and composite categories (those which have sub-categories). Thus, for the present study, we selected 51 categories that comprise of self-references (I, we, you, shehe), emotion (posemo, negemo, anxiety, anger), health and body (feel, body, health, sexual), psychology (focus present, focus future, swear) and other life aspects (work, leisure, home, money). We applied these lexicons to all tweets posted by a user on a particular day, resulting in a 51-dimensional frequency vector.

#### 2.3.4. Effect Estimation

**Figure 1:**
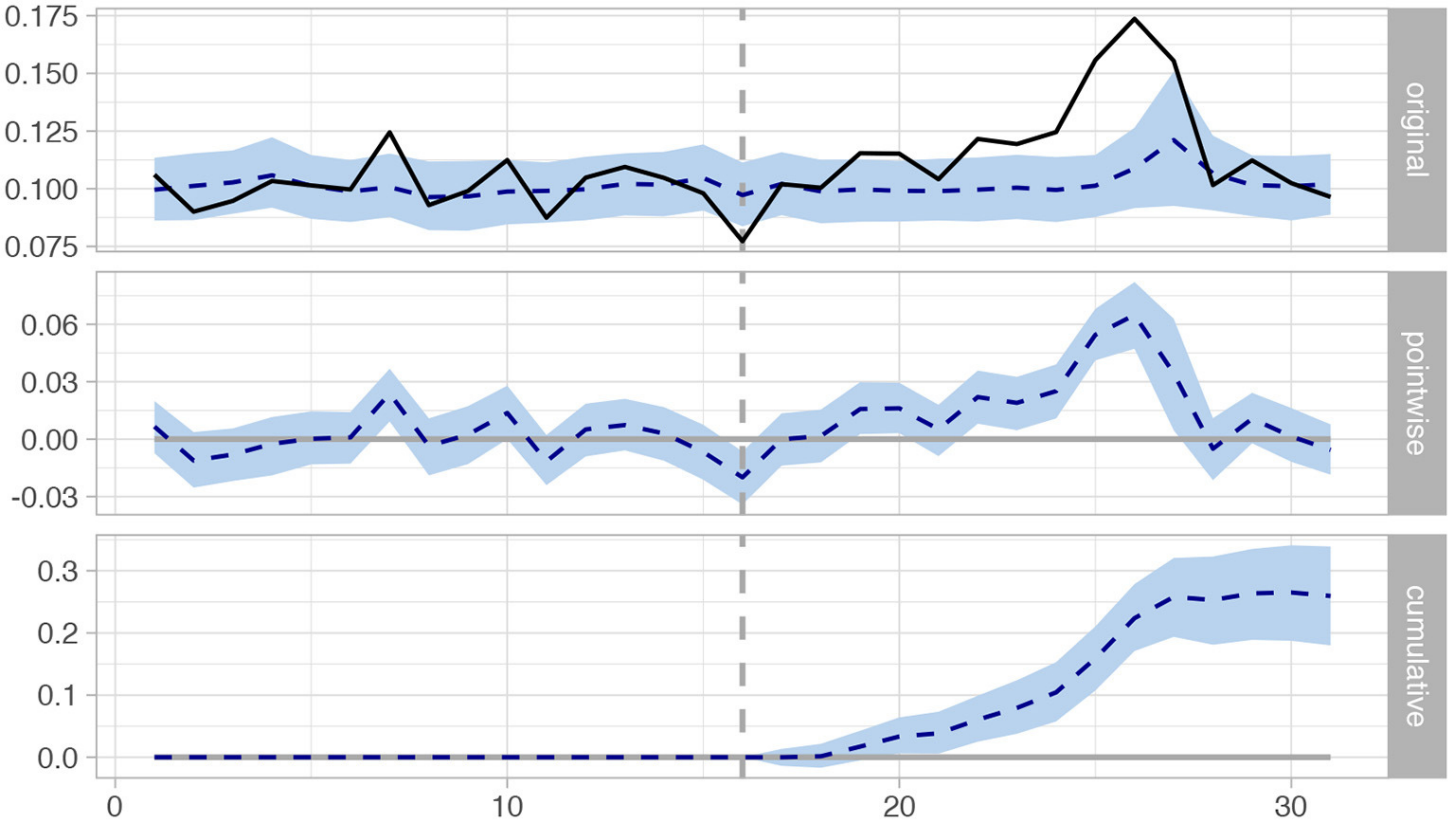
Time series in causal impact analysis for Female category, top: observed tweet rate (solid) and baseline (dashed), middle: difference between the two, bottom: cumulative effect after intervention.

The above modeling results in a time series for each user, which we can examine in order to determine whether there have been significant changes in the user's self-expression, operationalized using the LIWC word categories, after the user interacted with NEDAwareness content. Specifically, we employed *Causal Impact* analysis package (38) which takes time series data as an input, and attempts to estimate the effect of some intervention that happened at a particular point in time. This method compares the changes between a response time series (the target users) and a set of control time series (baseline). Given these two series, the method constructs a Bayesian structural time-series model that builds a prediction of the time series if the intervention had never occurred, and compares it to the actual outcome (38). Figure 1 provides an example: in the top plot the tweet rate for the *Female* LIWC category is represented as a solid line, and a baseline tweet rate as a dashed line. The middle plot shows the difference between observed data and the baseline. Finally, the third shows the cumulative effect of the intervention (shown in gray dashed vertical line), which we can see is positive. The package also provides confidence intervals and a *p*-value. We use *P* < 0.05, or *P* < 0.001 with the Bonferroni correction, as a thresholds in this study.

#### 2.3.5. Content Analysis

For the linguistic categories shown to have a significant change after the intervention, we use text processing to examine the themes present in the associated content. For each category, we find the tweets containing one or more words from this category, and tokenize them, as in the causality analysis. We then compute odds ratio (OR) (39) of words being used by one (female) and not the other (male) gender. Words that have a higher OR score are examined as more indicative of the peculiar topics mentioned by that gender around the category of interest. For these words, we read a sample of tweets and explore the context in which they were mentioned.

## 3. Results

### 3.1. Volume

**Figure 2:**
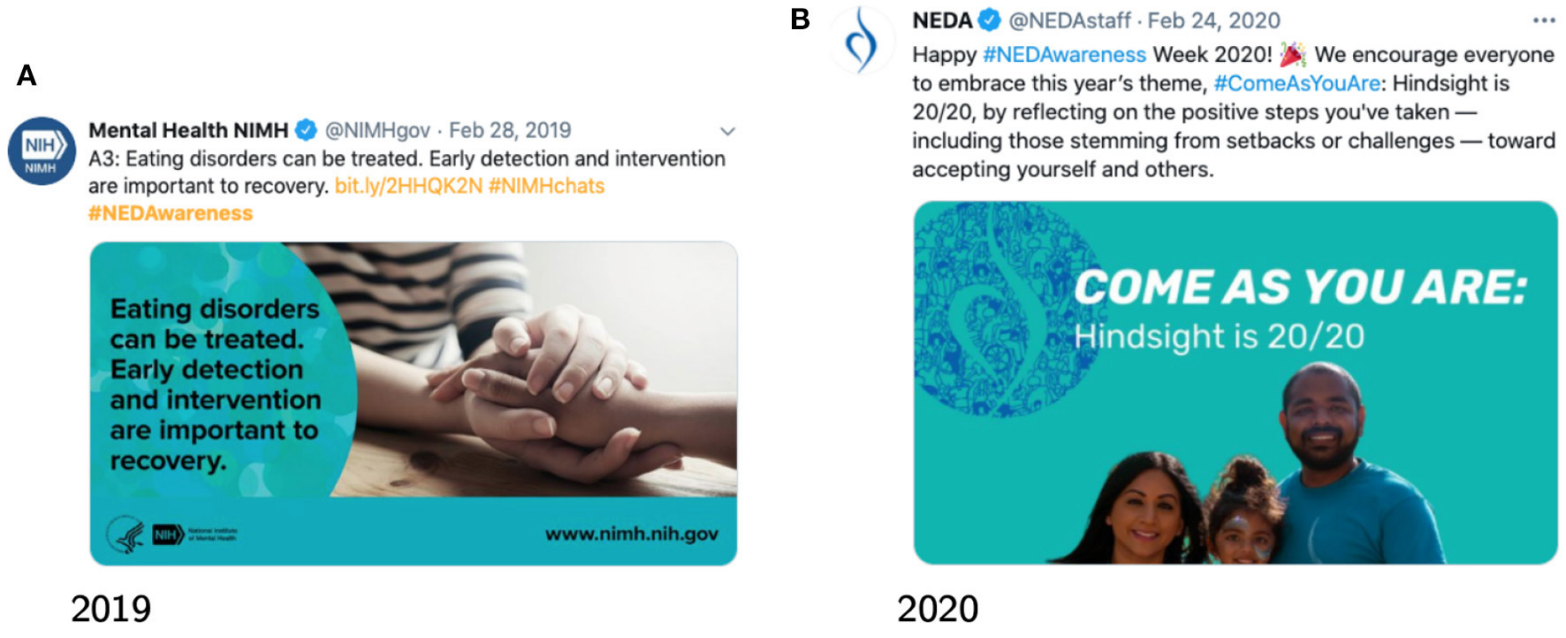
Example posts during the NEDAwareness campaigns.

**Figure 3:**
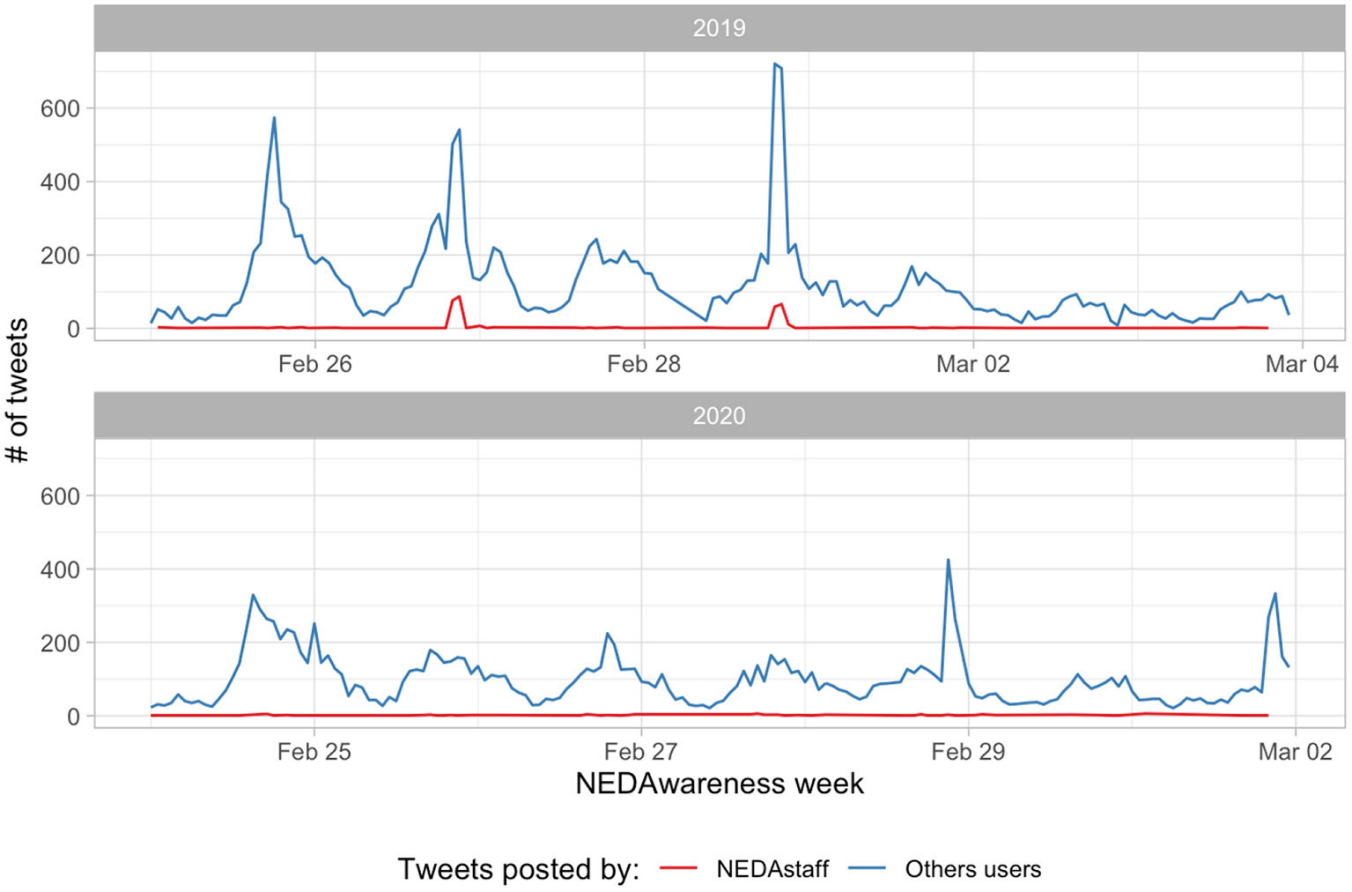
Tweets per hour during the NEDAwareness week during 2019 and 2020.

Figure 2 shows two posts exemplifying the content that was disseminated during these campaigns. The 2 years had the “Come as you are” theme, but with slightly different emphasis. In 2019 the campaign was encouraging for people from a plurality of ages, races, genders and gender identities to engage with the message and share their story. In 2020, the emphasis was on the reflection of the steps taken by those struggling with eating disorders and sharing their journeys publicly. Figure 3 shows the hourly number of tweets captured during the two campaigns. We find similar daily periodicity, however 2019 shows peaks not achieved by 2020 campaign, including for the content produced by the NEDAstaff account.

### 3.2. Reach

Overall, we find that the 2019 campaign had more tweets and retweets (19 K) than the 2020 one (16 K). Out of the accounts captured here (both originating and retweeting content) have been detected to be mostly female at 43.0% (8363/19432), male only at 17.6% (3416/19432), with the rest unknown 39.4% (7653/19432) in 2019 and female at 38.2% (6289/16463), male at 17.9% (2953/16463), and unknown at 43.9% (7221/16463) in 2020.

The potential audience of these tweets differs drastically between the years. Table 2 shows the 20 accounts with the largest number of followers who have retweeted NEDA content in both years. The account with the most reach that participated in both campaigns is @instagram, having about 36 million followers both years. More mainstream media companies have also participated: @MTV, @MTVNEWS, @WomensHealthMag, @MensHealthMag, and @TeenVogue in 2019, and @gmanews (Philippines), @CNNnews18 and @NYDailyNews in 2020. In 2019, additional reach was provided by accounts affiliated with governmental institutions, including The National Institute of Mental Health (@NIMHgov) and U.S. Department of Health & Human Services (@HHSGov), as well as the Human Rights Campaign (@HRC). As we can see from the table, fewer large institutional accounts participated in the 2020 campaign, making for a smaller pool of potential audience of the material.

**Table 2:** Accounts retweeting NEDAwareness content, ranked by number of followers (in thousands, K) during the campaigns of 2019 and 2020.

**2019**		**2020**	
**Username**	**# Followers**	**Username**	**# Followers**
instagram	36,665 K	instagram	35,993 K
MTV	15,499 K	gmanews	5,533 K
MTVNEWS	5,160 K	parentsmagazine	4,763 K
WomensHealthMag	4,581 K	CNNnews18	4,342 K
MensHealthMag	4,516 K	GMA	3,707 K
TeenVogue	3,340 K	inquirerdotnet	2,938 K
inquirerdotnet	2,792 K	sadierob	1,871 K
Ginger_Zee	2,340 K	DZMMTeleRadyo	1,332 K
Pinterest	2,337 K	NIMHgov	1,163 K
Jimparedes	1,751 K	womenshealth	932 K
harpersbazaarus	1,677 K	NYDailyNews	736 K
seventeen	1,359 K	AlvaroAlvaradoC	652 K
NIMHgov	1,153 K	SELFmagazine	507 K
womenshealth	936 K	nutribullet	442 K
HRC	811 K	Xiaxue	374 K
HHSGov	754 K	MentalHealthAm	333 K
dosomething	750 K	TrevorProject	290 K
ABC7NY	653 K	WCVB	287 K
teddyboylocsin	646 K	TWLOHA	278 K
Allure_magazine	576 K	raphablueberry	254 K

**Figure 4:**
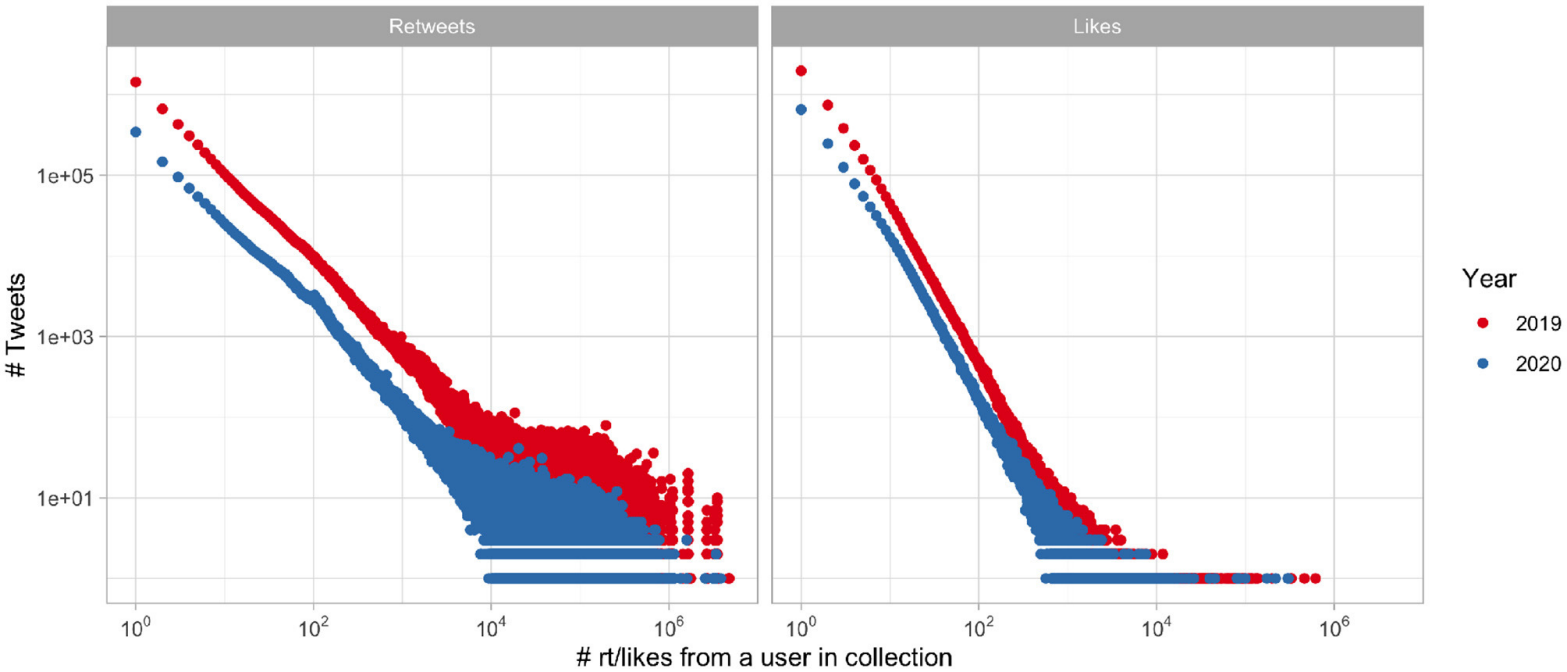
Distribution of tweets having certain number of retweets (left) and likes (right), log scale.

Further, in Figure 4 we show the retweet and like statistics of the content posted during the two campaigns. Not only do we see the decreased volume of the 2020 campaign, compared to 2019, we can also observe the lesser reach of the posted tweets. The average number of retweets of NEDA's content was 56.7 in 2019, whereas in the following year it was 38.6, we find the change to be significant using independent sample Student's *t*-test with Welch approximation to the degrees of freedom (*t* = 7.4, CI = 0.95, *P* < 0.001).

This change seems to have come from the popular accounts, since the median number of retweets changed only from 22 to 20, though the change is still significant using the Mann-Whitney U test (*W* = 4, 942, 067, CI = 0.95, *P* < 0.001).

In 2019 the average number of followers of the retweeting accounts averaged 3,610 (median = 333), signifying potential viewers per retweet of NEDAwareness content. In the following year, 2020, the average number of potential viewers dropped to 1,551 (median = 301). However Student's t-test shows that these means are not statistically different (*t* = 1.5, CI = 0.95, *P* = 0.06), pointing to the existence of a few large outliers in 2019 which are absent in 2020.

**Figure 5:**
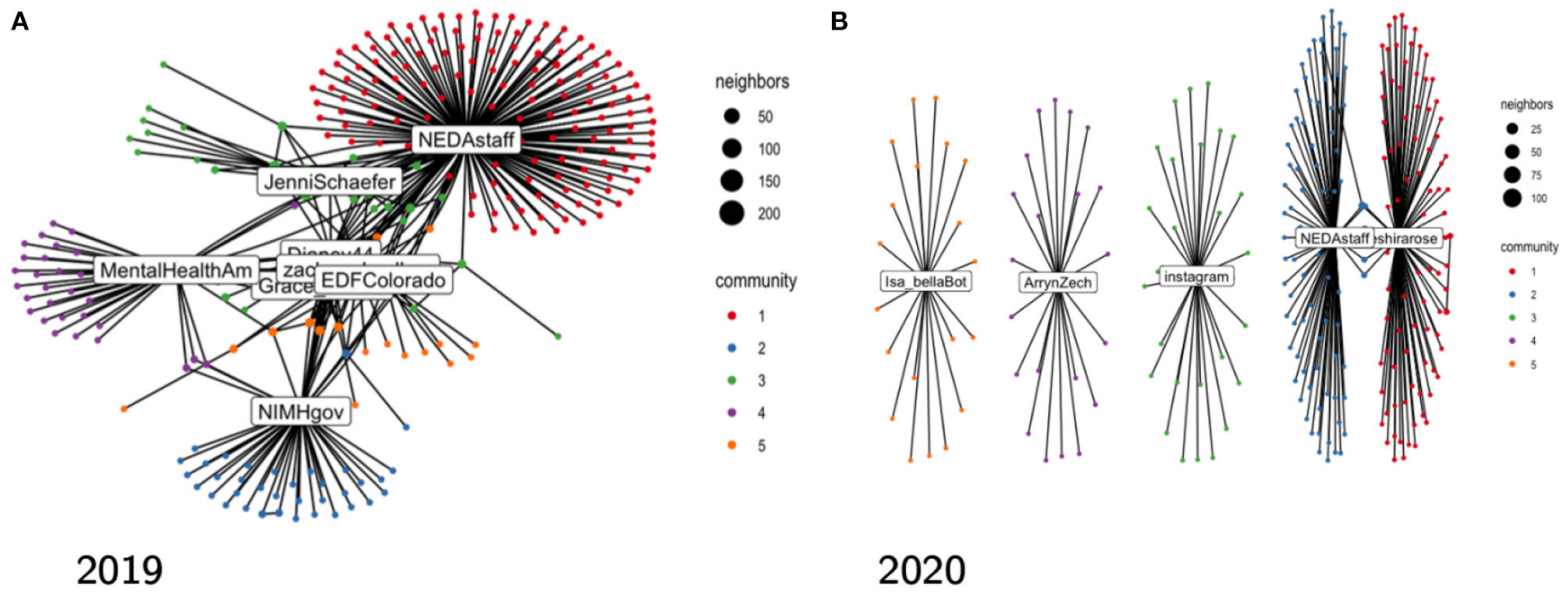
Top 5 communities in the retweet network, as identified using the Walktrap algorithm.

Figure 5 shows the five largest communities of the retweet networks in both years, as identified using the Walktrap algorithm (40, 41). We observe a well-connected giant connected component (GCC) in 2019 where governmental agencies, influencers, and the NEDAstaff accounts reinforce each other's message by retweeting. The top retweeted accounts include @NIMHgov and @MentalHealthAm (Mental Health America, a nonprofit organization), despite more popular generic accounts like @MTV participating, indicating that more topically relevant accounts may produce a wider reach. In 2020, we observe the opposite: a collection of stars, with only a few common pieces of content being shared. The top two most prominent users in the GCC are @NEDAStaff and @theshirarose (a self-described eating disorder psychotherapist). Note that, even though @Instagram account has several orders of magnitude more followers, it does not produce an outsized retweeting activity.

### 3.3. Behavioral Impact

**Figure 6:**
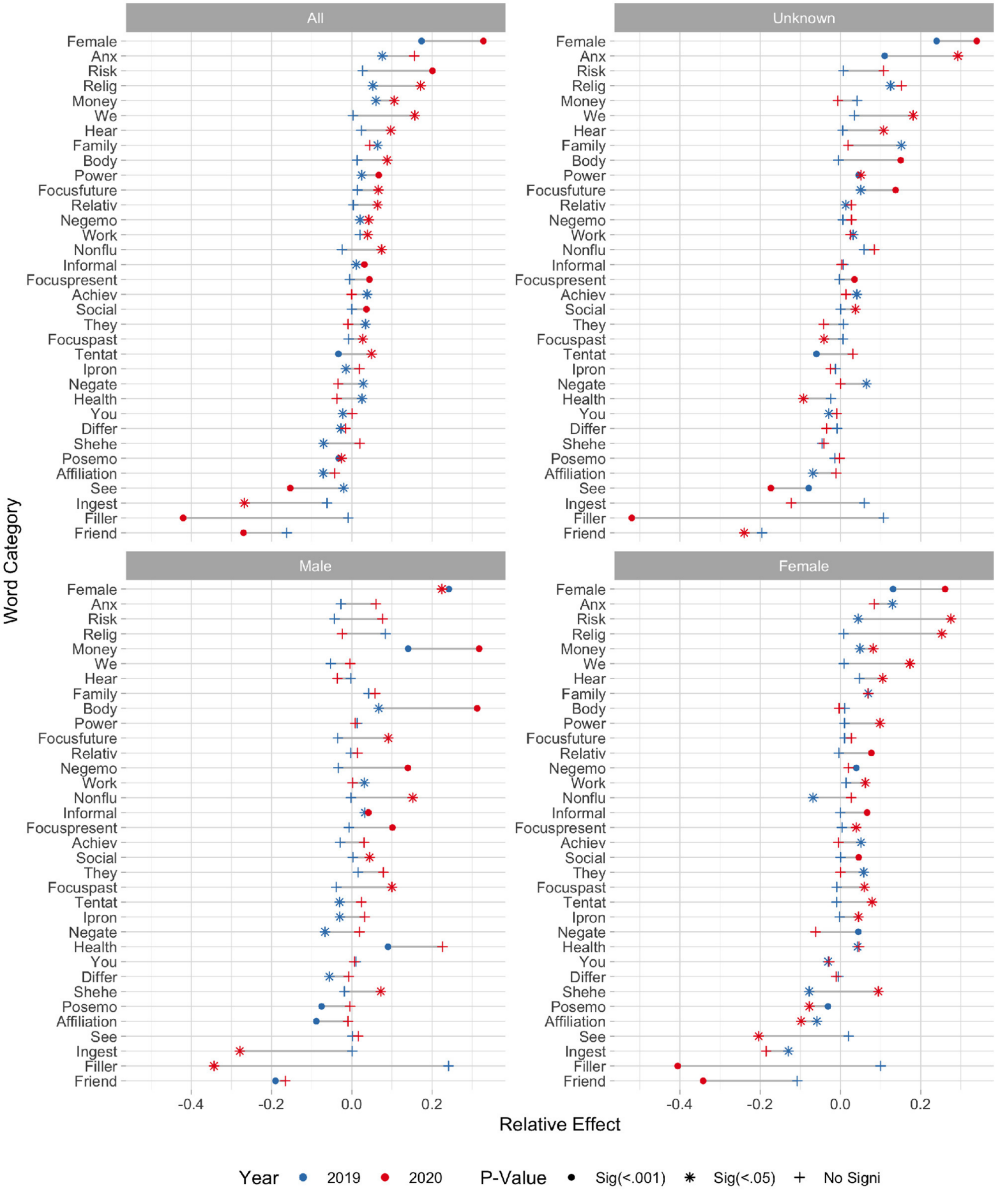
Relative effect of interaction with NEDA content upon users' use of LIWC categories in 2019 and 2020 campaigns. The *p*-values are encoded in marker: solid at *p* < 0.5 and cross for non-significant at 0.05.

Figure 6 shows the relative impact of users interacting with the NEDAwareness content during the 2019 (blue) and 2020 (red) campaigns on the content of their tweets. We show only the effects of at least 1% which are significant at *P* < 0.05 in the overall dataset in either year (not gender disaggregated). Thus, for these LIWC categories, users who have interacted with the NEDA content have changed the way they tweeted after the intervention beyond the changes in the general trend. These effects, however, are not uniform across the genders, as can be seen in the gender-specific panels (titled Unknown, Male, and Female). We show three levels of significance: at *P* < 0.05 marked with a star, *P* < 0.001 with a dot, and non-significant with a plus. The more strict *p*-value shows significance after we apply Bonferroni correction (due to multiple comparisons) to the significance level. Out of 34 categories, only 9 significantly change in both years (on gender disaggregated data), whereas others change significantly only in one of the years, although the direction of change is often the same. Overall, we observe stronger impacts on the expressions during the 2020 campaign than the 2019 one, also with the effects being stronger for the users identified as female. Below, we discuss the most significant changes in language that were detected in these campaigns.

#### 3.3.1. Changes in Language

As the figure shows, the *Female* category has shown some of the most profound change, in both years. This category contains words such as *women, she, her*, etc. For example, the following tweet talks about the trans woman identity and emphases the word *women*: “*rt (USER): trans women are women. trans women are women. trans women are women. trans women are women. trans women are women. trans women are..*..” When we consider all users, in 2019 the *Female* category showed an increase of +17% (95% interval [+11%, +23%]) and in 2020 an increase of +33% ([+23%, +43%]), meaning the users who have interacted with the NEDAwareness content were significantly more likely to mention female-related words than the control group. This effect is strong not only for user accounts identified as female, but also for male and unknown gender. Due to timing particularities, the annual International Women's Day occurs about a week after NEDAwareness campaign, on March 8, and is often marked by marches for women's rights, as well as more personal congratulations. Despite not being directly connected to eating disorders, this topic is favored by the users who have interacted with NEDAwareness campaign, pointing to a heightened awareness of this holiday, and possibly of the social issues it focuses on.

**Figure 7:**
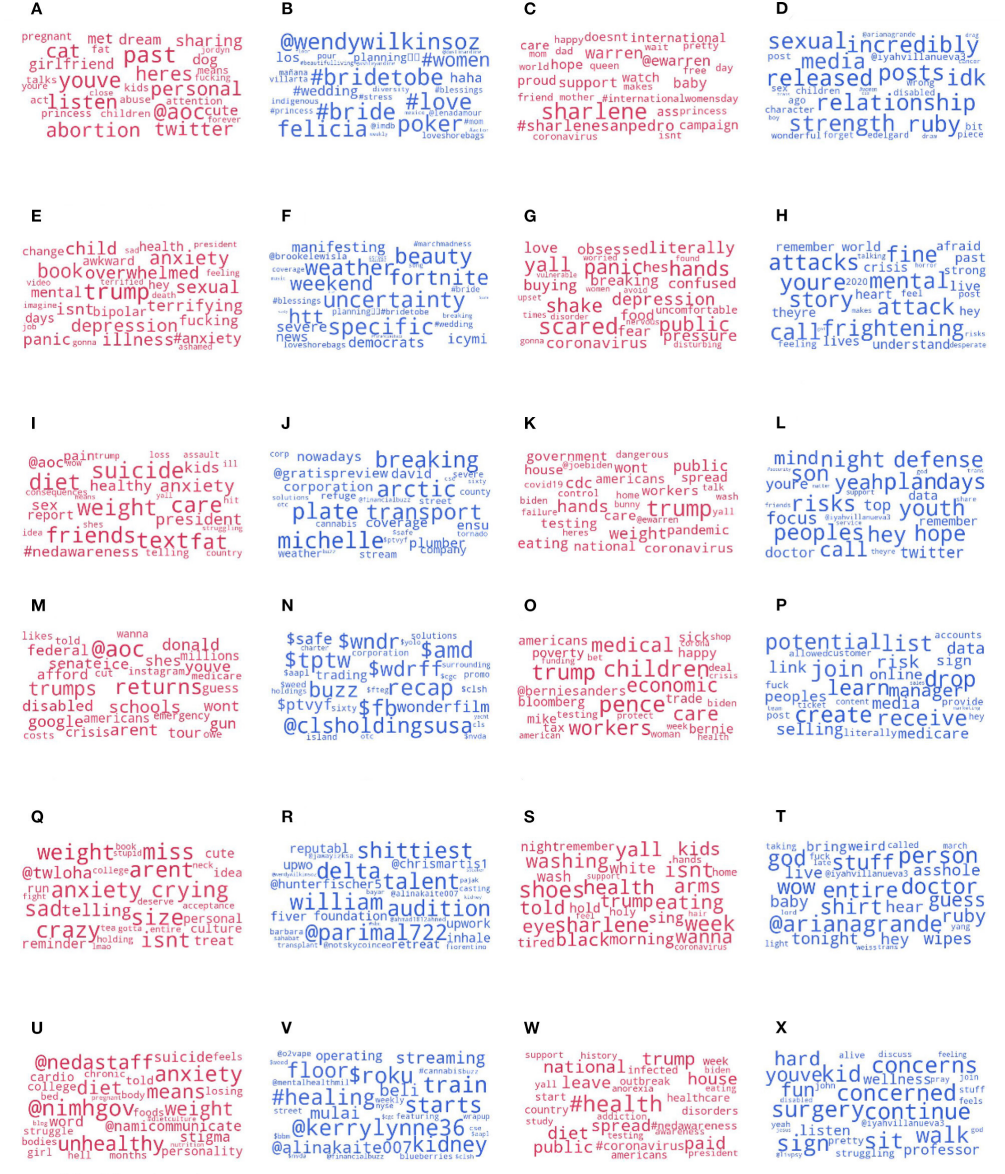
Top 30 words in categories, by gender and year. **(A)** Female: Female 2019, **(B)** Female: Male 2019, **(C)** Female: Female 2020, **(D)** Female: Male 2020, **(E)** Anxiety: Female 2019, **(F)** Anxiety: Male 2019, **(G)** Anxiety: Female 2020, **(H)** Anxiety: Male 2020, **(I)** Risk: Female 2019, **(J)** Risk: Male 2019, **(K)** Risk: Female 2020, **(L)** Risk: Male 2020, **(M)** Money: Female 2019, **(N)** Money: Male 2019, **(O)** Money: Female 2020, **(P)** Money: Male 2020, **(Q)** Body: Female 2019, **(R)** Body: Male 2019, **(S)** Body: Female 2020, **(T)** Body: Male 2020, **(U)** Health: Female 2019, **(V)** Health: Male 2019, **(W)** Health: Female 2020, and **(X)** Health: Male 2020.

Next, we focus on users who interacted with NEDAwareness content, and examine the most prominent words associated with the *Female* LIWC category posted immediately after this interaction. To do this, we compute odds ratio (OR) between words in tweets by female users and male users. Figures 7A–D shows such keywords which are more likely to be posted by female users (OR>1) and by male users (OR <1), in both years. We find that female users tend to more often mention politically-oriented topics, including *abortion* and the US Representative Alexandria Ocasio-Cortez (@*aoc*) in 2019, and the US Senator Elizabeth Warren (@*ewarren*) in 2020. Surprisingly, male users mention *#bride #poker* in 2019 and *relationship* and *sexual* in 2020. The 2019 bride-related tags for male users concern a viral tweet by an actress Brooke Lewis Bellas, and poker a tweet about famous female poker players. The 2020 ones stem from a viral tweet about a character in Disney's Mulan remake describing him as a “bisexual legend,” and another a critique of the LGBTQ+ community when it concerns bisexual women dating men. Thus, we find that male accounts who interacted with NEDAwareness content are more likely to discuss LGBTQ+ topics.

Instead, the category of *Anxiety* is affected differently both between the years and the genders. Meant to capture feelings of anxiety, this category includes words such as *risk, stress, upset* and *worry*. In 2019, both female and unknown gender users significantly increased their use of this category, and in 2020 only the unknown category. Male users did not change their usage of this category beyond the control in either year. Figures 7E–H shows most prominent words posted by users of each gender around the category of Anxiety. In both years, female users mention *depression*, and especially *anxiety* in 2019, while male users mention *uncertainty* and *frightening* and also *fortnite* game in 2019. Overall, we find more symptoms of depression and anxiety mentioned by female users including feeling *overwhelmed, shake*, and *panic*. Some of these were associated with an abbreviation ODAAT, which stands for 1 Day at a Time – which is a drug and alcohol recovery program, and an eponymous TV series. Interestingly, female users are more likely mention *coronavirus* in 2020, possibly showing a higher sensitivity to ongoing news (at that point only around 100 cases were recorded in the United States).

We find the category of *Risk* to be increased for the female users (and not other groups) in both years. This category includes words such as *apprehension, beware, concern*, and *danger*. In 2019, the use of this category by female users increased by +4% (95% interval[+1%, +8%]), and in 2020 by even greater +28% (95% interval [+12%, +44%]). From Figures 7I–L we see that, for female users, the category is most associated with *suicide, weight* and *diet* in 2019, and with *Trump, weight* and *pandemic* in 2020. One of the most retweeted posts talks about the constant belief that “*if I lose weight, I will be more attractive and fit in”*, critiquing the body shape obsession instilled in young women. On the other hand, the tweets retweeted by male users often involve promotion of apps, global warming, and politics (some of which are, again, about LGBTQ+ issues). Again, it is interesting to note that the COVID pandemic does not figure in the male tweets as prominently in 2020.

A few categories stand out when related to the accounts identified as male: *Money* and *Body*. Both years show significant elevation in mentioning of the two word categories. Figure 7 shows the top money-related and body-related terms mentioned by males and females. Whereas, female accounts continue talking about politics and schools, the male accounts focus on tickers (tags starting with $, for instance *$amd* stands for the stocks of Advanced Micro Devices company) in 2019 and around politics, economy and jobs in 2020. In 2020, we find a popular tweet claiming “*the idea is to create a virus that spreads to everyone in order to boost hand sanitizer profits.”*

Unfortunately, the *Body* category often matches with profanity, capturing heartfelt tweets from male accounts about companies (some especially disliked in 2019 were Upwork and Delta), and invocations to God when talking about politics and increasingly global pandemic in 2020.

Health category increased in 2019, but did not change significantly in 2020, perhaps because of already strong interest in the pandemic. To check whether health topic was indeed affected by the COVID pandemic, we use a list of relevant terms (covid*, *coronavirus, covid-19*) and track the proportion of COVID-related tweets posted by each group. Indeed, during the span of our study, the prevalence of COVID topic substantially increased both in the target group (*z* = 48.2, CI = 0.95, *P* < 0.001) as well as in the control group (*z* = 70.3, CI = 0.95, *P* < 0.001).

Most of such tweets mentioned words related to the health topic such as viruses, nurses, doctors, health, wellness, contagions, hospitals, etc. Returning to the larger topic of *Health*, we find that the female users are more likely to mention official accounts, such as the one associated with the National Institute of Mental Health (NIMH) and the National Alliance on Mental Illness (NAMI), and in 2020 one of the most popular tweets retweeted by female users was about the cost of healthcare during the pandemic.

The health-related tweets posted by male users, on the other hand, were more likely to advertise “detox” cleanses and promote kidney donation in 2019, although in 2020 one of the most popular tweet catching the attention of male users was about concern over the spread of coronavirus.

On the bottom of the Figure 6 we find LIWC categories which were used less by the users who have interacted with the NEDAwareness content, compared to the control. First note that, compared to categories which have increased, there are relatively few that have decreased in use. Though, notably, within these we find the *Ingest* and *Friend* categories, which have decreased in 2020. As it is difficult to ascertain why the users who have interacted with NEDAwareness content post less about eating and friends than the control, we speculate that the increased attention to COVID-19 in early March may have contributed to this. We also point to the small, but statistically significant decrease in the use of *Positive emotion* (*Posemo*) words, especially by the female accounts. This finding complements the above-mentioned increase in anxiety and risk-related topics.

## 4. Discussion

In this study, we illustrate the application of causal impact analysis to the behavior change monitoring around a health intervention on a social media platform. Social media has long been recognized as an important outlet for those dealing with mental health challenges as a place to seek advice, share experiences, and receive support (42). Monitoring self-expression around eating disorders is instrumental in understanding the motivations, realities, and social interactions that may both exacerbate or alleviate mental distress of people using online tools. Recent studies of such self-expression have examined most of the major social media websites, including Twitter, Facebook, and Reddit, with LIWC becoming the standard tool for text analysis in psychological research (43). Here, we not only monitor the impacts of a public health intervention on the behavior these social media users, we do so over two campaigns in order to examine the stability of the results.

It is important to mention that, we aim to capture the effects of the health awareness campaign run by NEDA. We worked together with NEDA to compile a list of hashtags they were using in the promotion, and “tagging,” of their post. Thus, we are not interested in the overall health awareness, or ED awareness, discussion on social media. In fact, we attempt to capture some of it in the baseline collection, which includes eating disorders vocabulary. There might have been a potential change in the kinds of hashtags that were used for this topic between 2019 and 2020, but a change in the vocabulary between the 2 years would preclude a comparison in the impact of the NEDA campaign compared to a consistent baseline.

We find that the reach and effect of the campaigns varies substantially between 2019 and 2020. Not only were there fewer tweets posted on 2020, the engagement with the campaign content in terms of retweets fell from 56.7 in 2019 to 38.6 in 2020. Network analysis has also shown less coordination between the most influential users, displaying several independent star clusters in 2020, instead of a complex giant connected component of 2019. This illustrates the importance of the *centrality* of users trying to promote a message on social networks, as defined in the influence maximization literature. Intuitively, in order to reach the most people, those promoting the message must not only be connected to many others, but these others should also have large social networks to which they can propagate the message (44). Thus, building a highly interconnected network of influential accounts prior to the campaign would improve the reach of the material. This insight adds structural component to the latest insights on the use of cultural elements to boost the reach of health campaigns (45).

The two campaigns also differed in the self-expression impact on the users who have interacted with their content. Although the campaign in 2020 had fewer tweets and engagement, it produced a more statistically significant change in behavior for the users in our study. It is possible that smaller campaigns reach those already interested in the topic, or dedicated to further promoting it, so that there is a more pronounced difference from the baseline. It is still unclear whether these campaigns reach those in need of an intervention, or whether, as has been recorded in other domains (46), they are “preaching to the choir.” To measure the change in awareness, in the next campaigns we suggest running surveys to assess the knowledge and attitudes of the target audience, as have been previously done for cancer prevention (47) and mental health (48). However, social media data can be used to compile a more extensive profile of the reached users in order to estimate a view of their history with eating disorders and coping mechanisms they have attempted (49, 50).

The fact that the Twitter users retweeting the campaign material are more female, and the strongest behavior change for those interacting with the campaign material is in their use of words in the *Female* category, reflects the fact that women are more likely to report eating disorders, including binge eating and fasting (51). Also, previous studies have shown that women tend to have more food avoidant beliefs (52), have higher body dissatisfaction (53) and have a higher healthcare utilization rate (54) than men, which may suggest they would be more active around these topics on social media. We also find that, in the context of womanhood, female users engaged in the campaign were likely to post about other health topics, including abortion and pregnancy, and to mention female politicians. Although the campaign did not emphasize public policy, our data shows that there is an interest in public health policy. Ongoing efforts, such as the Public Engagement in Health Policy Project in Canada (55) and the England Patient and Public Participation policy in NHS (56) may take advantage of social media as a platform for engaging with the public on health issues.

Further, a substantial part of the user sample in the campaigns also includes accounts identified as male (around 18% both years). There have been calls in the past to study mental health and eating disorders in men (57, 58), especially since there may be a greater association with substance abuse (59). Social media provides an opportunity to study men's relationship to eating disorders, their self-expression and community engagement, and their responses to health intervention campaigns.

Another major theme in the post-intervention behavior change revolves around the expression of anxiety and risk, especially by the users identified as female. Despite the supportive nature of the campaign's materials, we find the users continuing to discuss topics around diet, suicide, and depression, and especially in 2020 the ongoing coronavirus outbreak, which was declared by the WHO a pandemic on 11 March 2020. The public discussion of mental health issues and other stressors has been linked to a *psychological openness* that has been recorded in women who seek help from mental health professionals (60). During the pandemic, depression and anxiety has been associated with the use of social media in China (61), and it has been suggested that social media use may be a coping mechanism (62). To understand the long-term effects of emotional expression on social media, we urge the investment in longitudinal studies of its use in the context of mental health and eating disorders.

This study adds to a recent drive to quantify the engagement with online health awareness campaigns, specifically those promoted on Twitter. Previous studies have focused on the direct engagement with the content, such as categorizing the content mentioning the hashtag #MyTipsForMentalHealth (inspirational posts were retweeted the most) (20), as well as general awareness of the medical topic [such as mentions of various cancers around the cancer awareness months (63)]. Highly localized campaigns could be matched to outpatient data, such as the Bell Let's Talk campaign in Canada, which showed to be temporally associated with an increase in outpatient mental health utilization (64). The present study expands these approaches by monitoring the topical shifts in social engagement after the intervention, potentially capturing concerns, observations, and self-expression not captured within the direct interactions with the NEDAwareness campaign materials. Future work in coupling these observations with healthcare utilization, if such data can be made available, or using surveys [such as in Lyson et al. (65)] would provide further information on the health-related behaviors and beliefs of the campaign's audience.

Although complementary to the traditional survey-based methods, social media studies suffer from several limitations, which we mention here. First, when observing self-expression online, only thoughts and feelings the users chose to express at the time can be captured, which may be strategically composed to project a public persona (66). Still, many mental health studies have shown that social media is a valuable outlet and source of support for its users (43). Second, the demographics of the populations online are often unclear, with tools providing only an approximate view (around 40% of our data had “unknown” gender label), whereas others were identified using name matching, a heuristic that could be easily fooled by a spoofed profile. Note that, we are not able to detect when a user impersonates another gender, or simply puts a wrong name in the description. Thus, we are able to capture self-descriptions of the Twitter users, potentially disfavoring those not disclosing their gender for any number of reasons (privacy concerns, fluid gender identity, etc.) Further studies in technology usage will allow for a more precise estimates of message exposure (67). Third, linguistic tools, even standard and validated ones such as LIWC, often fail to capture the full context of the topics shared online, and sometimes provide faulty matches. For instance, in our study the swearwords were often matched with the LIWC *Body* category, resulting in a detection of significant behavior change, especially for the users identified as male. Qualitative analysis, which we have performed here by sampling tweets containing words of interest, is thus necessary. Moreover, the linguistic resources fail to capture the visual content users share, and which has been shown to be significant in the study of anxiety and depression (68) and eating disorders (69).

Finally, mental health research deals with potentially vulnerable populations, and whereas in this work only the largest accounts were revealed and example tweets rephrased as much as possible for de-identification, privacy is an ongoing concern. To limit the exposure of the individuals involved, the data will be made available to other researchers in anonymized fashion, and in accordance with EU General Data Protection Regulation (GDPR).

## Data Availability Statement

Derived data supporting the conclusions of this study are available upon request from the corresponding author.

## Author Contributions

All authors listed have made a substantial, direct, and intellectual contribution to the work and approved it for publication.

## Funding

The authors acknowledge support from the Lagrange Project of the Institute for Scientific Interchange Foundation (ISI Foundation) funded by Fondazione Cassa di Risparmio di Torino (Fondazione CRT).

## Conflict of Interest

The authors declare that the research was conducted in the absence of any commercial or financial relationships that could be construed as a potential conflict of interest.

## Publisher's Note

All claims expressed in this article are solely those of the authors and do not necessarily represent those of their affiliated organizations, or those of the publisher, the editors and the reviewers. Any product that may be evaluated in this article, or claim that may be made by its manufacturer, is not guaranteed or endorsed by the publisher.

## Footnotes

^1^The diet and health related keywords are as follows: *edmeme, edrecovery, effyourbeautystandards, embraceyourbody, everybodyisbeautiful, exercise, fasting, fatacceptance, fatactivism, fatjustice, fatloss, fatstudies, fitspo, fuckdietculture, gainingweightiscool, haes, healthateverysize, intermittentfasting, intuitiveeating, loseweight, nofatshaming, nondiet, nourish, nutrition, osfed, proana, recoveryposse, ribspo, sanctuarynutrition, starving, theantidietplan, thighgap, thin, thinspiration, thinspo, wakeupweightwatchers, weight, weightbias, weightinclusive, weightloss, weightstigma, wellbeing, allbodiesaregoodbodies, anabuddy, anamia, anorexia, anorexiarecovery, bingeeating, bodiposi, bodyacceptance, bodyconfidence, bodyimage, bodyimagemovement, bodyliberation, bodypositive, bodypositivity, bodyshaming, bonespo, bopo, bopowarrior, bulimia, calories, diet, dietculture, dieted, dieting, diets, ditchthediet, eatingdisorder, eatingdisordermeme, eatingdisorderssuck*.
