# Supplementary material for: Behavior Change Around an Online Health Awareness Campaign: A Causal Impact Study
Source: Front Public Health. 2022 Jun 23;10:857531. doi: 10.3389/fpubh.2022.857531 (PMC9260224; doi:10.3389/fpubh.2022.857531)
Supplement: Supplementary file 1 [file Data_Sheet_1.PDF]

1.Appendix 1

We provide source data for wordclouds (Figure 7). Each table contains data about different categories. We provide data by years and genre of the nick account. The odds Ratio is in OR Column. Female and Male counts are in f and m columns.

Body: Female 2019				Body: Male 2019			
word	OR	f	m	word	OR	f	m
size	3.253	57	8	william	0.104	6	33
anxiety	3.155	49	7	delta	0.133	19	75
miss	3.141	55	8	@parimal722	0.144	7	27
crazy	3.092	48	7	talent	0.149	7	26
crying	2.965	46	7	audition	0.149	7	26
weight	2.962	87	14	shittiest	0.156	8	28
arent	2.956	40	6	reputabl	0.156	8	28
sad	2.902	45	7	@chrismartis1	0.156	8	28
isnt	2.84	89	15	foundation	0.168	12	38
telling	2.839	44	7	@hunterfischer5	0.168	9	29
@twloha	2.812	38	6	inhale	0.177	6	19
reminder	2.74	37	6	@notskycoinceo	0.191	10	28
treat	2.668	36	6	retreat	0.202	7	19
personal	2.595	35	6	fiver	0.202	7	19
cute	2.563	65	12	fiorentino	0.212	7	18
culture	2.523	39	7	casting	0.212	7	18
run	2.523	39	7	@alinakaite007	0.212	7	18
idea	2.523	34	6	upwo	0.212	7	18
acceptance	2.467	43	8	upwork	0.212	7	18
holding	2.467	43	8	barbara	0.212	7	18
college	2.451	33	6	transplant	0.216	8	20
book	2.411	42	8	@jawayizksa	0.219	9	22
neck	2.411	42	8	bayar	0.221	6	15
deserve	2.379	32	6	pajak	0.221	6	15
tea	2.334	36	7	sahabat	0.227	8	19
entire	2.334	36	7	@wendywilkinsoz	0.229	9	21
stupid	2.334	36	7	@ahmad1812ahmed	0.229	9	21
fight	2.313	54	11	kidney	0.231	10	23
gotta	2.307	31	6	#sbc	0.231	10	23
lmao	2.307	31	6	speaker	0.235	6	14

Body: Female 2020				Body: Male 2020			
word	OR	f	m	word	OR	f	m
shoes	2.41	36	7	@arianagrande	0.208	7	19
week	2.084	39	9	person	0.236	9	21
isnt	2.084	35	8	doctor	0.245	7	16
sharlene	1.968	33	8	god	0.28	6	12
health	1.894	39	10	entire	0.325	19	31
arms	1.86	24	6	stuff	0.331	6	10
washing	1.841	52	14	shirt	0.331	6	10
yall	1.824	48	13	wipes	0.347	7	11
eating	1.78	40	11	ruby	0.378	7	10
told	1.711	22	6	guess	0.39	23	31
wanna	1.693	25	7	wow	0.396	15	20
eye	1.613	64	20	baby	0.405	6	8
black	1.563	38	12	tonight	0.409	10	13
trump	1.562	26	8	asshole	0.416	15	19
kids	1.562	23	7	hey	0.423	12	15
morning	1.562	20	6	live	0.423	12	15
white	1.515	31	10	hear	0.426	8	10
sing	1.488	19	6	bring	0.446	11	13
wash	1.436	145	52	weird	0.446	11	13
night	1.42	29	10	@iyahvillanueva3	0.455	6	7
hold	1.42	29	10	fuck	0.455	6	7
tired	1.413	18	6	called	0.455	6	7
remember	1.406	26	9	taking	0.455	6	7
holy	1.402	34	12	march	0.459	14	16
home	1.389	39	14	light	0.463	7	8
hands	1.387	262	98	yang	0.463	7	8
coronavirus	1.379	89	33	late	0.474	30	33
support	1.367	20	7	weiss	0.48	11	12
hair	1.367	20	7	trans	0.52	20	20
feel	1.359	59	22	lord	0.52	10	10

Health: Female 2019				Health: Male 2019			
word	OR	f	m	word	OR	f	m
@nimhgov	4.301	58	6	@kerrylynne36	0.05	6	71
anxiety	3.843	142	18	starts	0.067	8	67
unhealthy	3.38	52	7	$roku	0.068	8	66
@nedastaff	3.298	83	12	train	0.073	6	48
means	3.294	70	10	#healing	0.081	6	43
weight	3.183	186	29	kidney	0.089	10	62
diet	3.115	249	40	floor	0.091	21	122
@namicommunicate	3.061	41	6	@alinakaite007	0.1	53	274
stigma	3.061	41	6	beli	0.105	22	111
word	3.061	41	6	mulai	0.113	31	143
suicide	3.028	88	14	streaming	0.113	31	143
personality	2.934	45	7	operating	0.115	6	30
cardio	2.934	45	7	@mentalhealthmil	0.116	12	56
college	2.934	45	7	#cannabis	0.117	19	86
told	2.903	90	15	street	0.119	33	144
struggle	2.87	44	7	weekly	0.132	13	53
losing	2.843	38	6	blueberries	0.132	6	26
months	2.83	60	10	featuring	0.135	13	52
foods	2.83	60	10	@o2vape	0.14	13	50
girl	2.824	82	14	nyse	0.141	7	28
feels	2.778	48	8	wrapup	0.141	7	28
body	2.745	214	39	$bbm	0.141	7	28
chronic	2.722	79	14	@financialbuzz	0.143	6	24
bed	2.721	47	8	cse	0.143	40	145
hell	2.697	36	6	buzz	0.15	9	33
bodies	2.697	36	6	$clsh	0.156	10	35
#dietculture	2.679	41	7	$weed	0.159	13	44
pregnant	2.658	124	23	$aapl	0.159	23	76
blog	2.624	35	6	$nvda	0.16	15	50
nutrition	2.613	173	33	$cgc	0.163	7	24

Health: Female 2020				Health: Male 2020			
word	OR	f	m	word	OR	f	m
#health	4.23	85	9	sign	0.093	6	36
trump	2.703	65	11	concerns	0.156	6	21
national	2.506	50	9	walk	0.213	9	22
paid	2.408	48	9	continue	0.216	29	67
diet	2.395	38	7	sit	0.229	6	14
spread	2.316	32	6	concerned	0.283	14	25
house	2.316	32	6	kid	0.286	6	11
leave	2.278	50	10	surgery	0.312	6	10
public	2.251	118	25	youve	0.312	6	10
#coronavirus	2.233	49	10	fun	0.315	8	13
#nedawareness	2.211	44	9	hard	0.332	22	33
outbreak	2.105	29	6	professor	0.337	10	15
disorders	2.089	84	19	wellness	0.344	6	9
healthcare	2.088	33	7	listen	0.344	6	9
country	2.074	37	8	pretty	0.357	7	10
week	2.072	117	27	struggling	0.357	7	10
start	2.035	28	6	stuff	0.36	10	14
infected	2.026	32	7	@iyahvillanueva3	0.368	8	11
americans	2.019	36	8	john	0.382	6	8
president	2.014	40	9	alive	0.404	27	33
yall	2.014	40	9	feels	0.421	11	13
study	1.965	27	6	discuss	0.427	19	22
addiction	1.91	34	8	yeah	0.429	20	23
support	1.883	45	11	pray	0.43	6	7
awareness	1.876	41	10	join	0.43	6	7
testing	1.842	29	7	feeling	0.43	6	7
history	1.824	25	6	god	0.43	6	7
anorexia	1.812	58	15	disabled	0.43	6	7
eating	1.728	129	36	@livpsy	0.43	6	7
biden	1.719	27	7	jesus	0.43	6	7

Money: Female 2019				Money: Male 2019			
word	OR	f	m	word	OR	f	m
@aoc	9.253	93	9	recap	0.053	6	128
returns	8.012	56	6	$tptw	0.103	6	66
trumps	6.83	110	15	$amd	0.113	9	86
schools	5.575	50	8	@clsholdingsusa	0.119	6	57
arent	4.919	39	7	$fb	0.123	7	63
donald	4.637	32	6	buzz	0.13	8	67
google	4.515	77	16	wonderfilm	0.132	14	111
shes	4.382	48	10	$wdrff	0.133	64	477
youve	4.303	34	7	$wndr	0.136	7	57
ice	4.153	37	8	$safe	0.136	7	57
gun	4.075	28	6	$ptvyf	0.136	24	180
federal	4.067	61	14	trading	0.138	8	63
senate	4.057	32	7	corporation	0.14	19	139
disabled	4.057	32	7	promo	0.142	14	103
wont	4.044	36	8	$aapl	0.145	9	67
afford	3.935	47	11	island	0.146	14	100
tour	3.935	43	10	solutions	0.148	7	52
americans	3.935	39	9	sixty	0.152	10	70
millions	3.859	50	12	$weed	0.153	13	89
crisis	3.811	30	7	$clsh	0.166	14	88
guess	3.811	30	7	holdings	0.166	25	153
emergency	3.794	26	6	surrounding	0.169	9	57
wanna	3.738	37	9	otc	0.169	9	57
instagram	3.682	100	26	$fteg	0.169	9	57
cut	3.654	51	13	$nvda	0.172	68	392
costs	3.641	73	19	$yolo	0.176	11	66
told	3.64	36	9	cls	0.176	11	66
likes	3.623	80	21	$cgc	0.179	11	65
owe	3.513	49	13	charter	0.179	9	54
medicare	3.513	24	6	yacht	0.18	14	81

Money: Female 2020				Money: Male 2020			
word	OR	f	m	word	OR	f	m
pence	3.321	53	8	join	0.088	13	87
children	3.32	41	6	drop	0.098	12	72
trump	3.152	73	12	list	0.152	7	28
workers	2.712	48	9	create	0.258	6	14
medical	2.608	32	6	receive	0.276	8	17
economic	2.49	44	9	potential	0.32	10	18
care	2.457	70	15	manager	0.352	6	10
@berniesanders	2.45	30	6	learn	0.352	6	10
americans	2.397	38	8	risk	0.368	13	20
happy	2.336	37	8	selling	0.368	9	14
sick	2.293	57	13	data	0.368	9	14
trade	2.158	38	9	media	0.383	8	12
poverty	2.151	34	8	medicare	0.383	8	12
mike	2.151	34	8	sign	0.387	20	29
bernie	2.133	26	6	peoples	0.387	6	9
bloomberg	2.133	26	6	link	0.387	6	9
tax	1.976	49	13	fuck	0.387	6	9
testing	1.975	24	6	online	0.387	6	9
deal	1.975	24	6	literally	0.399	12	17
biden	1.967	31	8	post	0.399	12	17
woman	1.905	30	8	provide	0.407	13	18
protect	1.896	23	6	accounts	0.414	11	15
american	1.896	23	6	customer	0.414	8	11
bet	1.881	33	9	allowed	0.414	8	11
shop	1.881	33	9	hey	0.43	6	8
week	1.855	56	16	content	0.43	6	8
health	1.845	89	26	ticket	0.452	8	10
funding	1.778	44	13	team	0.452	8	10
corona	1.77	31	9	sales	0.452	8	10
crisis	1.745	40	12	marketing	0.46	14	17

Anxiety: Female 2019				Anxiety: Male 2019			
word	OR	f	m	word	OR	f	m
trump	4.331	75	7	specific	0.039	7	92
book	3.232	77	10	weather	0.04	7	90
anxiety	3.143	431	62	beauty	0.04	7	90
depression	2.828	123	19	uncertainty	0.04	7	90
sexual	2.798	42	6	htt	0.048	8	84
child	2.735	95	15	weekend	0.082	8	49
illness	2.651	63	10	fortnite	0.093	8	43
overwhelmed	2.603	39	6	manifesting	0.12	13	52
terrifying	2.449	42	7	severe	0.124	8	32
isnt	2.409	73	13	democrats	0.157	8	25
panic	2.384	67	12	icymi	0.192	7	18
#anxiety	2.379	46	8	news	0.192	7	18
mental	2.372	155	29	@brookelewisla	0.192	7	18
fucking	2.369	51	9	#marchmadness	0.192	7	18
bipolar	2.342	35	6	#wedding	0.192	7	18
health	2.26	113	22	#blessings	0.192	7	18
days	2.227	43	8	#bride	0.192	7	18
awkward	2.195	52	10	#bridetobe	0.215	8	18
change	2.187	47	9	planning	0.224	42	86
hey	2.164	37	7	loveshorebags	0.228	6	13
president	2.147	32	6	coverage	0.245	6	12
feeling	2.115	64	13	song	0.26	15	27
imagine	2.107	36	7	breaking	0.265	6	11
job	2.082	31	6	music	0.273	8	14
video	2.082	31	6	street	0.273	8	14
ashamed	2.05	53	11	@financialbuzz	0.273	8	14
gonna	1.993	34	7	$safe	0.293	8	13
death	1.993	34	7	cse	0.294	10	16
terrified	1.959	42	9	solutions	0.303	11	17
sad	1.952	29	6	sixty	0.315	8	12

Anxiety: Female 2020				Anxiety: Male 2020			
word	OR	f	m	word	OR	f	m
scared	2.92	55	11	mental	0.134	7	36
hands	2.444	42	10	youre	0.312	6	13
panic	2.399	68	17	fine	0.49	10	13
public	2.343	29	7	attacks	0.499	7	9
yall	2.32	25	6	frightening	0.52	9	11
shake	2.141	23	6	story	0.52	9	11
depression	1.717	21	7	attack	0.534	35	41
literally	1.695	18	6	past	0.546	6	7
pressure	1.686	26	9	understand	0.546	6	7
coronavirus	1.587	108	42	call	0.554	15	17
obsessed	1.533	26	10	live	0.554	7	8
buying	1.516	16	6	world	0.554	7	8
breaking	1.516	16	6	theyre	0.554	7	8
confused	1.489	30	12	crisis	0.561	8	9
love	1.489	30	12	remember	0.563	27	30
fear	1.458	55	23	afraid	0.567	9	10
hes	1.457	20	8	lives	0.576	11	12
food	1.427	15	6	hey	0.585	14	15
uncomfortable	1.427	15	6	strong	0.624	13	13
disturbing	1.427	15	6	heart	0.624	9	9
times	1.387	19	8	feeling	0.624	8	8
women	1.373	21	9	post	0.624	8	8
gonna	1.338	14	6	2020	0.624	8	8
disorder	1.326	16	7	feel	0.624	7	7
found	1.318	18	8	risks	0.624	7	7
worried	1.308	43	20	desperate	0.624	6	6
nervous	1.305	22	10	talking	0.624	6	6
upset	1.29	30	14	makes	0.624	6	6
avoid	1.249	59	29	horror	0.653	42	40
vulnerable	1.249	31	15	god	0.655	20	19

Female: Female 2019				Female: Male 2019			
word	OR	f	m	word	OR	f	m
past	3.101	49	7	#bridetobe	0.13	9	37
@aoc	2.906	40	6	#bride	0.131	8	33
youve	2.811	50	8	#love	0.142	13	48
abortion	2.425	43	8	@wendywilkinsoz	0.142	13	48
listen	2.409	33	6	poker	0.154	8	28
heres	2.324	88	18	felicia	0.162	16	51
cat	2.294	36	7	#women	0.165	6	20
personal	2.294	36	7	#wedding	0.172	7	22
twitter	2.274	54	11	haha	0.184	9	26
sharing	2.26	40	8	los	0.186	11	31
girlfriend	2.26	40	8	#blessings	0.194	24	63
met	2.255	49	10	#stress	0.201	16	41
dog	2.216	66	14	loveshorebags	0.202	10	26
dream	2.197	30	6	planning	0.203	8	21
cute	2.162	60	13	pour	0.203	8	21
pregnant	2.137	55	12	#princess	0.205	16	40
princess	2.115	80	18	ma±ana	0.209	7	18
act	2.095	37	8	@lenadamour	0.209	7	18
talks	2.055	28	6	@imdb	0.209	7	18
children	2.05	94	22	indigenous	0.209	7	18
attention	2.046	32	7	#mom	0.216	9	22
means	2.039	36	8	villarta	0.217	6	15
fat	2.026	48	11	diversity	0.223	8	19
abuse	2.026	48	11	#beautifulliving	0.225	14	32
kids	2.023	105	25	mexico	0.225	9	21
youre	1.996	196	48	#actor	0.233	7	16
fucking	1.985	75	18	@dustinardine	0.233	7	16
jordyn	1.984	35	8	@ashleyardine	0.233	7	16
forever	1.984	31	7	weekly	0.235	8	18
close	1.984	31	7	floor	0.235	8	18

Risk: Female 2019				Risk: Male 2019			
word	OR	f	m	word	OR	f	m
weight	3.578	238	34	arctic	0.058	6	62
text	3.53	53	7	plate	0.07	18	141
suicide	3.509	113	16	michelle	0.071	18	139
diet	3.399	51	7	breaking	0.078	16	113
friends	3.269	74	11	transport	0.078	16	112
care	3.262	105	16	nowadays	0.088	14	88
fat	3.09	64	10	ensu	0.088	25	153
anxiety	2.981	56	9	corporation	0.094	16	93
president	2.914	77	13	plumber	0.104	6	34
#nedawareness	2.876	43	7	@gratispreview	0.125	11	49
@aoc	2.853	59	10	david	0.136	12	49
sex	2.824	53	9	coverage	0.146	6	24
healthy	2.789	47	8	company	0.148	12	45
kids	2.726	98	18	weather	0.158	15	52
report	2.68	40	7	stream	0.183	6	19
pain	2.614	44	8	refuge	0.196	8	23
telling	2.614	39	7	street	0.211	16	41
consequences	2.498	42	8	cannabis	0.22	7	18
loss	2.256	236	54	county	0.224	11	27
shes	2.248	42	9	severe	0.224	8	20
hit	2.241	29	6	corp	0.229	6	15
country	2.234	46	10	tornado	0.229	6	15
idea	2.207	37	8	solutions	0.229	6	15
ill	2.19	66	15	$safe	0.229	6	15
trump	2.178	153	36	$ptvyf	0.229	6	15
assault	2.166	28	6	otc	0.232	11	26
wow	2.166	28	6	@financialbuzz	0.238	41	91
struggling	2.157	32	7	cse	0.244	6	14
yall	2.153	69	16	sixty	0.244	6	14
means	2.149	36	8	buzz	0.245	14	31

Risk: Female 2020				Risk: Male 2020			
word	OR	f	m	word	OR	f	m
trump	6.436	111	10	plan	0.176	6	24
hands	3.342	52	9	youth	0.233	9	26
weight	2.881	31	6	risks	0.252	9	24
public	2.634	70	16	night	0.283	22	50
cdc	2.61	28	6	peoples	0.294	6	14
wont	2.284	28	7	hey	0.315	9	19
eating	2.25	24	6	hope	0.354	8	15
workers	2.07	22	6	defense	0.374	27	46
coronavirus	2.055	194	59	son	0.377	11	19
care	2.026	44	13	days	0.387	7	12
government	2.005	34	10	yeah	0.393	9	15
testing	1.969	24	7	mind	0.397	11	18
pandemic	1.89	23	7	call	0.405	8	13
house	1.89	20	6	focus	0.431	67	98
spread	1.849	43	14	twitter	0.441	6	9
national	1.82	25	8	top	0.45	9	13
americans	1.82	25	8	youre	0.458	7	10
dangerous	1.772	44	15	doctor	0.472	11	15
home	1.764	41	14	remember	0.472	11	15
control	1.75	24	8	data	0.472	8	11
@joebiden	1.732	21	7	@iyahvillanueva3	0.484	9	12
talk	1.732	21	7	theyre	0.49	6	8
covid19	1.68	31	11	support	0.497	14	18
biden	1.68	23	8	share	0.503	19	24
wash	1.638	25	9	friends	0.504	7	9
yall	1.627	30	11	service	0.504	7	9
failure	1.546	26	10	god	0.524	14	17
heres	1.54	21	8	trans	0.525	9	11
bunny	1.536	38	15	matter	0.551	6	7
@ewarren	1.53	16	6	#security	0.555	14	16
